# Isolation and Characterization of *Gramineae* and *Fabaceae* Soda Lignins

**DOI:** 10.3390/ijms18020327

**Published:** 2017-02-04

**Authors:** Juan Domínguez-Robles, Rafael Sánchez, Eduardo Espinosa, Davide Savy, Pierluigi Mazzei, Alessandro Piccolo, Alejandro Rodríguez

**Affiliations:** 1 Chemical Engineering Department, Faculty of Science, University of Córdoba, Building Marie-Curie, Campus of Rabanales, 14014 Córdoba, Spain; z42doroj@uco.es (J.D.-R.); q92saser@uco.es (R.S.); a02esvie@uco.es (E.E.); 2 Centro Interdipartimentale di Ricerca sulla Risonanza Magnetica Nucleare per l’Ambiente, l’Agro-Alimentare ed i Nuovi Materiali (CERMANU), Via Università 100, 80055 Portici, Italy; davide.savy@unina.it (D.S.); pierluigi.mazzei@unina.it (P.M.); alessandro.piccolo@unina.it (A.P.)

**Keywords:** agricultural residues, alkaline extraction, spent liquors, soda lignin, physico-chemical properties, structural elucidation

## Abstract

Some agricultural residues such as wheat or barley straw, as well as certain fast-growing plants like *Leucaena leucocephala* and *Chamaecytisus proliferus*, could be used as raw materials for the paper industry as an alternative to traditional plants (eucalyptus, pine, etc.). In the present study, four types of lignin obtained from the spent liquors produced by the pulping processes using the abovementioned feedstocks were isolated and characterized. Lignin samples were acquired through an acid precipitation from these spent liquors. The characterization of the precipitated lignin samples were performed using a Fourier transform infrared spectroscopy (FT-IR) and both liquid- and solid-state nuclear magnetic resonance spectroscopy (NMR) to analyse the chemical structure, and thermogravimetric analysis (TGA) for determining the thermal properties. Additionally, chemical composition of lignin fractions was also measured. Even though they were of different botanical origin, all the studied samples except for wheat straw lignin had a similar chemical composition and thermal behaviour, and identical chemical structure. Wheat straw lignin showed a greater amount of Klason lignin and lower carbohydrate content. Furthermore, this lignin sample showed a higher thermal stability and significantly different cross-peak patterns in the 2D-NMR experiments. The molecular structures corresponding to *p*-coumarate (PCA), ferulate (FA) and cinnamyl aldehyde end-groups (J) were only detected in wheat isolated lignin.

## 1. Introduction

Lignocellulosic biomass are mainly formed of three structural components, cellulose, hemicellulosic polysaccharides and lignin, with other non-structural components in residual amounts. This biomass is an abundant source of raw materials that are becoming progressively important as a source of energy and chemical products [1].

Within the lignocellulosic biomass, cereal straws are an important source of raw material due to the world production of crops such as rice, wheat, barley, etc. [2]. The use of agricultural waste has a great ecological value in that it is an environmentally responsible means of consumption. Additionally, due to their structural characteristics, these cereal straws are suitable for producing pulp and paper [3,4]. Furthermore, there are also some leguminous species such as *Leucaena leucocephala* or *Chamaecytisus proliferus* that have been investigated for biomass and paper production [5,6,7]. These fast-growing non-woody species have been traditionally used as high-quality forage for ruminants but also can be an alternative source of pulp wood or energy production [7,8].

To achieve the maximum economic value of the whole lignocellulosic material (included agricultural residue), an integral exploitation should be taken into account. Potential industrial utilizations of the components of these lignocellulosic biomass include a large number of applications; (i) cellulose may be used for production of cellulosic pulps or bioethanol through different processes and transformations; (ii) hemicellulose may be exploited for production of xylo-oligosaccharides [9], or electricity and hydrogen by sequential fermentation [10]; and finally (iii) many compounds or applications could be developed using lignin such as active carbon [11] carbon fibre [12] or could be exploited as a raw material in the polymer industry [13].

However, only a small proportion of the lignin generated in the paper industry (i.e., 2% of the Kraft lignin) is exploited to develop new applications or for producing chemical compounds. The rest of this aromatic polymer is usually burned to obtain energy, after a previous step of black liquor concentration [14]. These data provide a promising scenario for lignin exploitation.

An important step to exploit the components of the lignocellulosic biomass is the fractionation method used [1]. For pulp and paper production, separation of cellulosic fibre is necessary. Different processes could be used for this purpose; among them, it is worth underlining Kraft or soda pulping processes, the latter being the one most used for delignification of agricultural residues [15]. Soda lignin is sulphur-free and also has a composition that is closer to the composition of natural lignin compared to Kraft lignin. Both attributes allow the use of this type of lignin directly without any purification step [16]. Therefore, the abovementioned applications could enhance the competitiveness of the pulp production, due to the high added value of the other components.

An important amount of effluent is generated during these processes. This liquid waste generally has a black or dark brown colour and, therefore, is called black liquor. Lignin is one of the main components of this liquor because during the pulping processes the aromatic polymer fraction is fragmented and dissolved from the raw material becoming part of such liquor [17]. Apart from lignin, a wide variety of compounds can be found in the black liquor, including those derived from lignin and other toxic compounds [18].

Lignin is a complex phenolic polymer synthesised mainly from three monomers, called *p*-coumaryl, coniferyl and sinapyl alcohols, which lead to *p*-hydroxyphenyl (H), guaiacyl (G) and syringyl (S) units, respectively [19]. This complex aromatic heteropolymer plays a major role in the support tissues of vascular plants and also protects them against pathogens.

For years now, numerous scientific works have focused on elucidating the structure of lignin [20,21,22,23]. However, a handicap is the isolation method used to obtain a chemically unchanged form of lignin [24]. In this sense, the isolation yield, composition and structure of lignins could vary depending on the extraction method used. Thus, the characteristics of the lignin are important to consider for future applications of this polymer [25].

**Figure 1:**
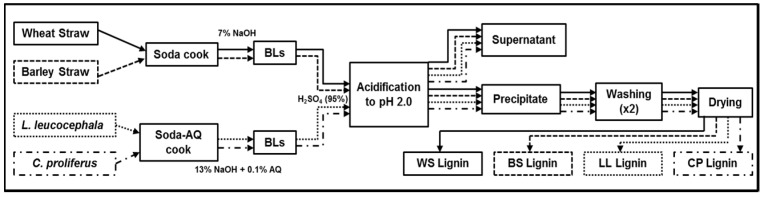
Scheme of lignin isolation. AQ, Anthraquinone; BLs, black liquors; WS, wheat straw; BS, barley straw; LL, *Leucaena leucocephala*; CP, *Chamaecytisus proliferus*.

In the present work, an alkaline extraction of lignin (AL) under mild and high-temperature conditions was used to extract lignin from cereal straws and leguminous plant species, respectively. The objective was to compare the lignin samples collected from both soda pulping processes using four types of feedstocks; two of them were agricultural residues coming from the family *Gramineae* (wheat and barley straw), and the other two were plant species that belong to the *Fabaceae* family (*Leucaena leucocephala* and *Chamaecytisus proliferus*). This knowledge is important to improve the exploitation of this interesting residue for chemical products and biomaterial production as well as for a source of energy (Figure 1).

## 2. Results and Discussion

### 2.1. Black Liquor Composition

The analysis of the black liquor composition is important given that lignin samples are extracted from it. The physicochemical properties of the untreated black liquor are shown in Table 1. The values of density and total dry solids content were higher in the case of *Fabaceae* plants, probably due to the greater severity of the pulping process used. Higher percentage of the reagent (sodium hydroxide) is required in this case (*Fabaceae* plants) to obtain cellulosic pulps with better qualities. Besides this, black liquor obtained from wheat straw showed a lower content of total dry solids compared with that obtained from barley straw. However, the proportion of ash over dry matter of these liquors using wheat straw (49%) was larger than that reported for the other three liquors (37%, 40% and 42% for barley straw, *Chamaecytisus* and *Leucaena*, respectively). These results are in agreement with the high content of ash (9.6%) and silica (7.3%) found in the wheat straw [26]. The content of lignin also increased with an increasing percentage of NaOH (from 7% to 13%), since the lignin percentage of the different raw materials was very similar (16.30%, 16.80%, 17.70% and 18.40% for barley straw, *Chamaecytisus*, wheat straw and *Leucaena*, respectively). The measurement of the black liquors’ conductivity showed that the greater percentage of NaOH used in the pulping process (legume species) increased the electrical conductivity.

The content of total dissolved carbohydrates in the obtained spent liquors ranged from 3.812 to 5.049 g/L. Once again, it was higher in the liquors in which *Fabaceae* plants were the raw materials used. These values are similar to those found in Kraft liquors (3.6–4.0 [18] or 3.56 g/L [27]) and slightly higher than those found in Organosolv liquors (3.2 g/L [28]), as is expected. The maximum amount of these dissolved polysaccharides was obtained with *Chamaecytisus*, mainly for the high content of xylose (2.624 g/L) and acetyl groups (1.514 g/L). Furthermore, the content of glucose (1.237 g/L) in the black liquor sample of *Leucaena* was much higher than that found in the other three samples and also in the abovementioned Kraft liquors. More information about these total dissolved polysaccharides is given in Table 1.

### 2.2. Lignin Fractions Characterization

#### 2.2.1. Yield and Chemical Composition

The yield and chemical composition of the precipitated fractions after the acidification of the black liquor samples are presented in Table 2. The greater amount of precipitate obtained using the fast-growing plants as raw materials was probably due to the higher temperature and NaOH percentage of the pulping process employed in these cases (from 100 to 150 °C and from 7% to 13% over dried material (o.d.m.), respectively). The large values of these two process variables (temperature and soda concentration) cause an increased amount of the raw material to be dissolved in the black liquor. Thus, a large amount of precipitate was achieved after the acidification of the spent liquors obtained from *Fabaceae* plants, since the acid precipitation is a non-selective process for lignin.

As a result of the larger amount of lignin in the black liquors of leguminous plants (Table 1), the lignin found in their precipitated fractions (13.95 and 14.66 g/L Black liquors (BL) for *Chamaecytisus* and *Leucaena*, respectively) was slightly higher than those obtained with gramineous (9.93 and 12.56 g/L BL for barley straw and wheat straw, respectively). However, the percentage of extraction was lower in the leguminous than in the cereal straws. The yield of the wheat straw soda lignin is above 100% (108.28%), which could be explained by the incorporation of carbohydrates’ degradation products in the lignin matrix, as is reported in the literature for soda pulping processes [29]. Furthermore, in other processes such as the ethanol-based organosolv process, the formation of “pseudo-lignin”, which is a product of lignin condensation with extractives, proteins and hemicellulose-derived products, has also been reported [30,31]. These products cannot be discriminated from native lignin by the analytical method used.

As can be seen in Table 2, the percentage of acid-soluble lignin (ASL) was larger in the lignin fractions obtained from fast-growing plants. ASL is mainly composed of low-molecular-weight degradation products and hydrophilic derivatives of lignin [32]. Thus, a temperature increase (from 100 to 150 °C), a larger amount of the reagent used (from 7% to 13% o.d.m.) and the use of 0.1% Anthraquinone (AQ) as catalyst increased the proportion of low molecular weight lignin. This is due to the depolymerisation of the lignin, after an increase in the cooking severity.

**Figure 2:**
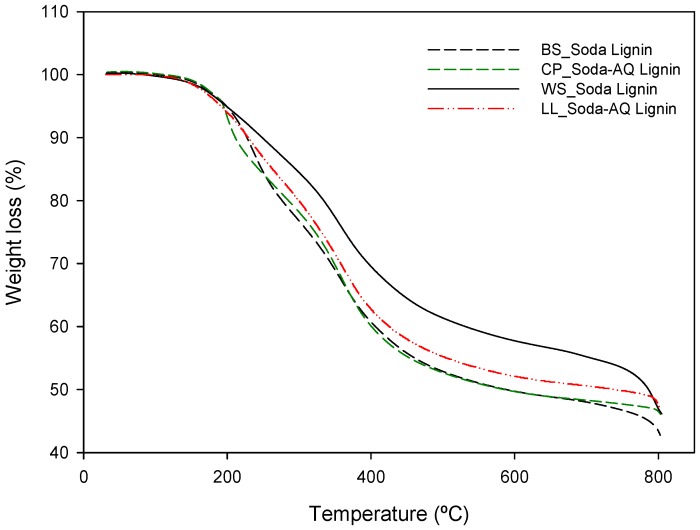
Thermogravimetric (TG) curves of lignin samples obtained from a thermogravimetric analysis (TGA). BS_Soda Lignin, CP_Soda-AQ Lignin, WS_Soda Lignin and LL_Soda-AQ Lignin refer to the lignin samples isolated from barley straw, *Chamaecytisus proliferus*, wheat straw and *Leucaena leucocephala*, respectively.

**Figure 3:**
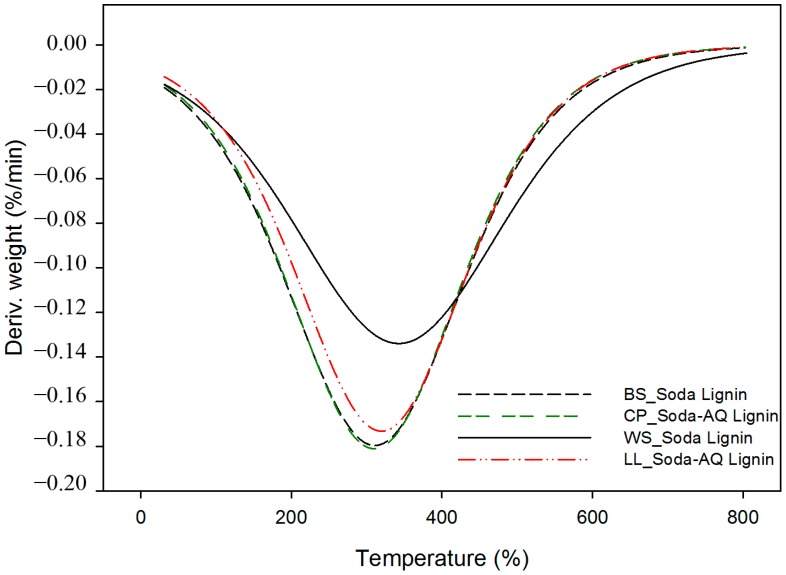
DTG curves of lignin samples obtained from a thermogravimetric analysis (TGA). BS_Soda Lignin, CP_Soda-AQ Lignin, WS_Soda Lignin and LL_Soda-AQ Lignin refer to the lignin samples isolated from barley straw, *Chamaecytisus proliferus*, wheat straw and *Leucaena leucocephala*, respectively.

The content of carbohydrates found in the wheat straw soda lignin (2.76% o.d.m.) is much lower than that found in the other three lignin samples (11.44%, 12.25% and 12.27% o.d.m. for *Leucaena*, *Chamaecytisus* and barley straw, respectively). These results are in accordance with the thermal properties presented in Figure 2 and Figure 3.

The ash content of the four lignin samples were relatively high (from 15.59% to 29.25% o.d.m.), even though these were conscientiously washed twice. This value was especially large for wheat straw soda lignin sample due to the raw material used, since wheat straw has a high amount of silica and other inorganic particles, as explained above. Finally, it is important to note that all the studied samples except wheat straw lignin had a similar chemical composition (Table 2).

#### 2.2.2. Thermal Behaviour

The thermal stability and degradation temperature of lignin samples are important parameters in order to study their applicability for biocomposite processing. Thermogravimetric (TG) curves, which indicate the weight loss of lignin samples in relation to the temperature of thermal degradation, and the first derivative of that curve (DTG), which shows the corresponding rate of weight loss, are presented in Figure 2 and Figure 3, respectively. In these curves two important temperature properties are analysed, T_onset_ and DTG_max_. The onset temperature (T_onset_) is expressed as the temperature at which the sample weight loss becomes more apparent. It was calculated by extrapolating the slope of the DTG curve in correspondence with the first local maximum in the second derivative TG curve (D^2^TG) and down to the zero level of the DTG axis [33]. DTG_max_ is the peak of the first derivative weight loss curve, which is the temperature of the maximal degradation rate.

Soda wheat straw lignin showed a T_onset_ at around 220 °C, which was slightly higher than that obtained from the other three raw materials (207, 208 and 209 °C for barley straw, *Leucaena* and *Chamaecytisus*, respectively). Furthermore, DTG_max_ for soda wheat straw lignin was also higher than the other three, as can be observed in Table 3. These differences could be explained by the chemical composition of the lignin samples (see Table 2). The lignin content of the precipitated fractions is higher in the soda wheat straw lignin, and also the amount of sugars of this sample is much lower than in the other three. In the lignocellulosic material, lignin, due to its chemical composition and structure (three kinds of benzene propane units), is heavily cross-linked and has a high molecular weight. Thus, this polymer is more stable and is difficult to decompose compared with the other two fractions, hemicellulose and cellulose [34]. This also indicates that a lower amount of sugars could improve the thermal stability of the lignin samples.

Figure 2 shows that, for the weight loss of 50%, the temperature was higher in soda wheat straw lignin (780 °C), followed by *Leucaena* (672 °C), barley straw (552 °C) and *Chamaecytisus* (548 °C). These results reveal that soda wheat straw lignin decomposes much less than the other three lignin samples, although the one obtained from *Leucaena* was less decomposed than the other two. In the four lignin samples, a large percentage of residues still remained at 800 °C under a nitrogen atmosphere (see Figure 2). This could be due to the complexity of lignin structures (i.e., high degree of branching) and the formation of highly condensed aromatic structure [35]. Thus, these samples are stable at high temperature.

#### 2.2.3. Chemical Structure

**Figure 4:**
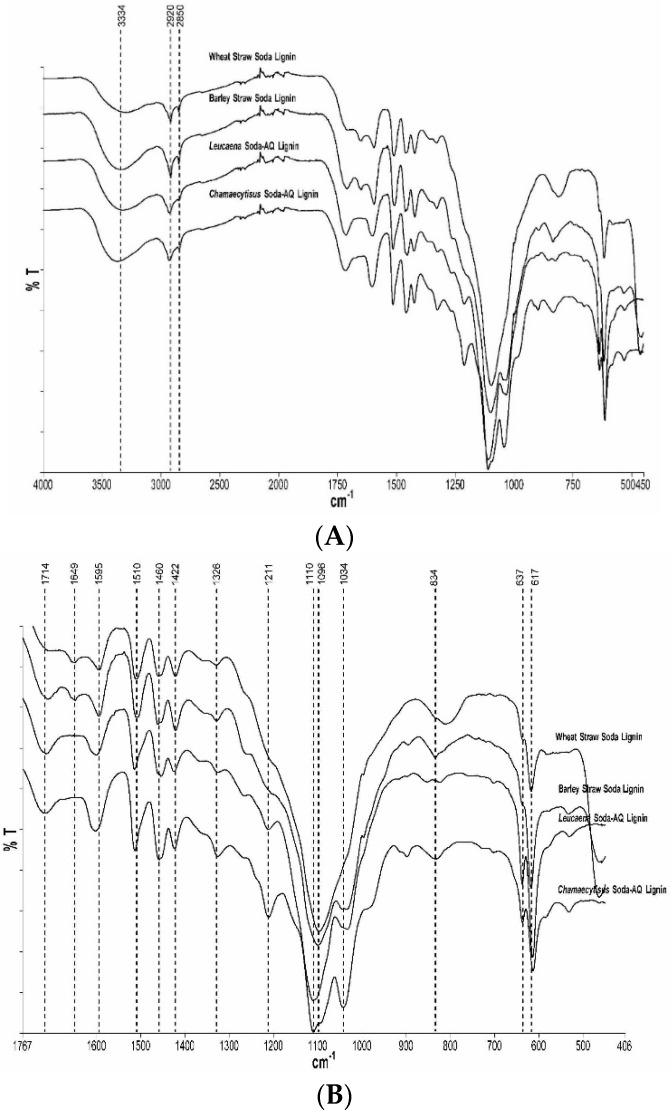
FT-IR Spectra of the four lignin samples (**A**); and a magnification of 1760–400 cm^−1^ region (**B**).

The chemical structure of the obtained lignin fractions was studied using attenuated total reflectance infrared Fourier transform spectroscopy (ATR-IR) and nuclear magnetic resonance spectroscopy (NMR). The ATR-IR spectra of the lignin samples were recorded in the 4000–450 cm^−1^ (Figure 4A) and a magnification of the 1760–400 cm^−1^ region is also presented in Figure 4B. Although the four lignin samples showed similar spectroscopic patterns, some differences were found in these soda lignin spectra related to the different lignocellulosic materials used.

A wide band centred at 3334 cm^−1^ corresponding to the aromatic and aliphatic OH groups is evident in all the obtained lignin samples. Absorbance of this band showed a lower intensity in the wheat straw soda lignin, indicating a lower content of hydroxyl group and thus showing a lignin structure less fragmented than the other three samples [18]. Bands located at 2920 and 2850 cm^−1^ are assigned to the symmetrical and asymmetrical C–H stretching vibrations of the methyl and methylene groups, respectively. Furthermore, a band at 1460 cm^−1^ is also related to the asymmetrical C–H stretching vibration. The bands at 1595, 1510 and 1422 cm^−1^ are assigned to aromatic ring vibrations of the phenylpropane units (lignin skeleton). These bands are presented in all of the studied lignin spectra and seem to be quite similar.

The band located at 1714 cm^−1^ is related to stretching vibrations of C=O bonds, in either ester linkages or carboxyl groups. A difference in the absorption intensity of this band was found between the wheat straw soda lignin and the other three samples. The higher intensity of this band may indicate the greater amount of carboxyl groups in hemicellulose; this fact confirms results found in the chemical composition of the lignin fractions (Table 2), where soda wheat straw lignin showed the lowest amount of carbohydrates. In that regard, the absorption band located at 1034 cm^−1^ is missed in the spectrum of wheat straw soda lignin; however, it is quite considerable in the other three lignin samples. This band is assigned to stretching vibrations of C-H bonds in the aromatic rings and C–OH bending in carbohydrates [36]. Once again, this point supports the different values of carbohydrates found in the chemical composition of lignin fractions (Table 2).

Minor differences in the lignin structure of *Gramineae* and fast-growing plants were found in the spectra. Bands at 1649 cm^−1^ assigned to conjugated carbonyl groups in the lignin were only detected in the two cereal straws. On the other hand, the absorption band at 1211 cm^−1^ related to the vibrations of guaiacyl rings were only found in the spectra of both fast-growing plants. However, the ^13^C-^1^H 2D-HSQC NMR spectra (a technique with greater accuracy) revealed the presence of Guaiacyl (G) units in all the obtained lignins (Figure 6B,D).

The bands at 1110 or 1096 cm^-1^ (assigned to C–H bending in plane of S units) and the band at 834 cm^−1^ (corresponding to C-H out of plane in positions 2 and 6 of S units and in all positions of H units) are typical of HGS type lignins [37]. Signal at 1326 cm^−1^ presented in all the spectra is assigned to C–O stretching vibrations of S units. Finally, the band at 617 cm^−1^ indicated the use of sulphuric acid to precipitate the four lignin samples since this signal was attributed to C–S stretching. All the assignments are based on previously published scientific papers [1,18,37,38,39].

#### 2.2.4. NMR Spectroscopy

**Figure 5:**
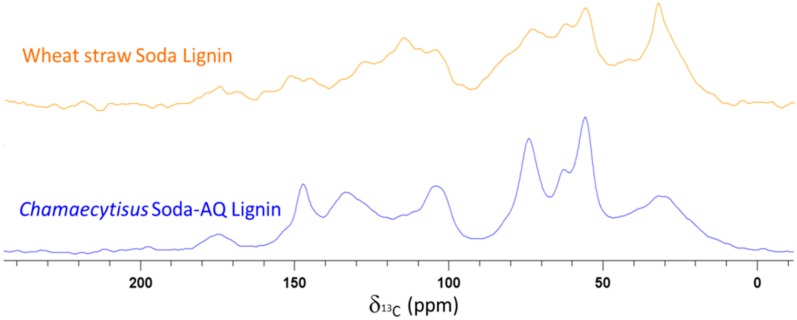
^13^C-CPMAS solid-state NMR spectra of lignin samples from wheat and *Chamaecytisus* lignin extracts. Spectra for lignin isolated from *Leucaena* and barley are shown in Figure S1 (Supplementary Materials), due to their close similarities with the spectrum of *Chamaecytisus* lignin.

**Figure 6:**
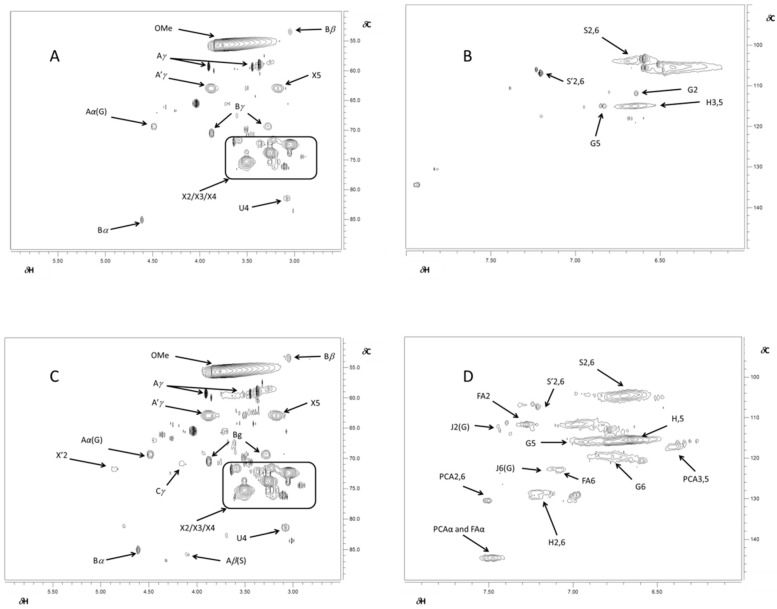
^13^C-^1^H 2D-HSQC NMR spectra of alkyl-oxidized (δC/δH 50–90/2.5–6.0 ppm) and aromatic (δC/δH 90–106/6.0–8.0 ppm) regions of lignins isolated from *Chamaecytisus proliferus* (**A**,**B**) and wheat (**C**,**D**), respectively. Spectra for lignins isolated from *Leucaena* and barley are shown in Figure S2 (Supplementary Materials), due to their close similarities with the spectrum of *Chamaecytisus* lignin. Labels refer to identified lignin sub-units (shown in Figure 7), whose signal assignment is reported in Table 5.

The ^13^C-Cross Polarization Magic Angle (CPMAS) solid-state NMR spectra of lignin samples from wheat and *Chamaecytisus* are shown in Figure 5. The spectra of lignins from *Leucaena* and barley are shown in Figure S1 (Supplementary Materials), since they were similar to those obtained for the *Chamaecytisus*-derived material. The peak around 32 ppm has been attributed to alkyl carbons, whereas that around 56 ppm was ascribed to the methoxy groups [40]. Moreover, the signal around 62 ppm was related to the lignin lateral chain and C5 in hemicellulose. Other peaks arising from the resonance of carbons in carbohydrate moieties can be found around 74 and 104 ppm, the latter of which could also be assigned to C2 and C6 of Syringyl monomer [41,42]. The peaks around 115 and 128 ppm were due to the C5 in Guaiacyl units and to both C2 and C6 in *p*-hydroxyphenyl units, while C1 for both Guaiacyl and Syringyl monolignols was identified around 133 ppm [43]. Other peaks attributed to the resonance of lignin carbons are those around 152 and 146 ppm, which were ascribed to the resonance of C3/C5 in etherified Syringyl units and of C3/C4 *non*-etherified Guaiacyl units, respectively [42]. Finally, the peaks around 175 ppm have been assigned carboxyl and ester groups from lignins and hemicellulose [44]. Despite the different nature of the agri-food residues used in this work, the lignin samples obtained after the alkali extraction and the acid precipitation from *Chamaecytisus*, *Leucaena* and barley were very close in structure and composition, as can be observed in Table 2 and Figure 5 and Figure 6. This can be a positive factor for the industry in that it permits us to achieve the same lignin pattern no matter the raw material used.

The integration of the ^13^C-CPMAS spectra allowed us to estimate the relative abundance of carbon nuclei resonating in different spectral regions (Table 4). Such carbon distributions were calculated only for lignins from wheat and *Chamaecytisus*, due to the similarities in the CPMAS spectra of samples from *Chamaecytisus*, *Leucaena* and barley extracts, as outlined above. Lignins from *Chamaecytisus* and wheat contained different hydrophobic moieties (alkyl and aromatic groups), whereas the relative amount of hydrophilic molecules (*O*-alkyl chains, carbohydrates, carbonyl and carboxyl groups) were found to be similar in the two materials (Table 4). The hydrophobicity (HB) and hydrophilicity (HI) were 42.2 and 57.5 for *Chamaecytisus* lignin and 46.5 and 53.6 for the wheat sample. Such indexes indicate the slightly more hydrophobic character of the lignin from wheat, as also suggested by the hydrophobicity Index, defined as the HB to HI ratio, which is significantly larger for the extract from wheat (0.87) compared to that from *Chamaecytisus* (0.79). The more hydrophobic nature of wheat lignin could mainly be attributed to the larger content of alkyl groups in wheat isolate with respect to the *Chamaecytisus* lignin, as suggested by the larger alkyl-related hydrophobicity index for wheat than for *Chamaecytisus* (Table 4).

Further details on lignin structure at a molecular level have been obtained by short-range correlation ^13^C-^1^H HSQC NMR experiments (Figure 6). Since cross-signals found in 2D-NMR experiments for lignins from *Chamaecytisus*, *Leucaena* and barley were similar (Figure S2), while wheat showed significantly different cross-peak patterns, we only report the 2D HSQC spectra for *Chamaecytisus*- and wheat-derived material. The related cross-peak attributions are reported in Table 5. The cross-signals in the range of δC/δH 50–90/2.5–6.0 ppm (Figure 6A,C) provided information on aliphatic oxygenated structures, i.e., lignin side chain and dimeric sub-units. The most abundant signal, resonating at 55.5/3.7 ppm, was related to lignin methoxy groups (OMe) [45]. The most present dimer was the β-*O*-4′ subunit, which could be identified by the resonance of a number of cross-peaks (Figure 6A,C). The C_γ_-H_γ_ correlations in either β-*O*-4′ (A) or in γ-acylated β-*O*-4′ substructures (A′) can be found around 59.3/3.2 and 3.9 ppm and 62.9/3.9 ppm, respectively [46], while the cross-peaks corresponding to the C_α_-H_α_ correlations of the same dimers were identified at 69.4/4.5 and 71.1/4.8 ppm. Finally, the signal at 85.9/4.1 ppm was assigned to the C_β_-H_β_ correlations for β-*O*-4′ linkage in Syringyl (S) units [47]. Moreover, the correlation for the C_β_-H_β_, C_γ_-H_γ_ and C_α_-H_α_ in the resinol (β-β) substructure were found for the cross peaks at 53.64/3.1, 70.6/3.3 and 3.9 and 85.1/4.6 ppm, respectively. The cross-signal at 70.9/4.2 was related to the occurrence of the phenylcoumaran (β-5′) dimer [44], which was present only in the wheat-isolated lignin (Table 5). The occurrence of residual carbohydrates was shown by the signals attributed to either xylopyranoside or 2-*O*-Ac-β-d-xylopyranoside sugars, the latter only being present in wheat lignin. Also, the 4-*O*-methyl-α-d-GlcUA (U_4_) could be identified (Figure 6A,C) [46]. Moreover, a close examination of the anomeric part of the HSQC spectrum (Figure S3) revealed the presence of other carbohydrates, namely the Methyl(1/4)-α-d-galacturonate (UGA_1_), (1/4)-β-d-Mannopyranoside (M_1_), and β-d-xylopyranoside+3-*O*-acetyl-β-d-xylopyranoside (X_1_-X′_1_) (Table 5) [1].

The aromatic spectral region (δC/δH 90–150/6.0–8.0 ppm) in the HSQC spectra revealed the occurrence of Guaiacyl (G), Syringyl (S) and *p*-hydroxyphenyl (H) units (Figure 6B,D), as expected from such raw biomasses [48]. The correlation for the C_2_-H_2_, C_5_-H_5_ and C_6_-H_6_ in G-units were found for the cross-peaks at 111.7/6.6, 115.6/6.7 and 119.8/6.8 ppm, respectively, while the short range correlation for C_2,6_-H_2,6_ resonance in etherified S units and oxidized (C_α_=O) phenolic syringyl units (S′) were found at 103.9/6.7 and 106.8/7.2 ppm, respectively [47]. Moreover, the ^13^C-^1^H correlations for the C_3,5_-H_3,5_ and C_2,6_-H_2,6_ in H-units were located at 115.1/6.7 and 129.0/7.2 ppm. Additional cross-peaks detected only in wheat lignin were related to *p*-coumarate (PCA) and ferulate (FA), whose resonances were found around 110.6/7.4, 122.9/7.1 and 130.3/7.5 ppm for FA_2_, FA_6_, PCA_2,6_ in the order. Moreover, the correlation for the C_α_-H_α_ for both FA and PCA molecules was found at 144.5/7.5 ppm, while the cross-peak around 117.1/6.4 ppm arose from the resonance of PCA_3,5_, Finally, the correlations between C_2_-H_2_ and C_6_-H_6_ in cinnamyl aldehyde end-groups (J) were found at 112.1/7.4 and 122.7/7.1 ppm, respectively [44]. These results show that this wheat straw lignin may be particularly interesting for the extraction of *p*-coumarate and ferulate to be used in the chemical industry as commodity chemicals [22].

## 3. Materials and Methods

### 3.1. Lignocellulosic Feedstocks

*Leucaena leucocephala* (Lam.) de Wit used in this work was harvested in Huelva (Spain) and provided by University of Huelva, Huelva, Spain. Samples of *L. leucocephala* were milled to pass through an 8-cm screen and the resulting wood chips were reduced again to pieces from 2 to 10 mm long. Also, in the case of *Chamaecytisus proliferus* samples were collected from experimental farms in the city of Huelva and provided by the same university. Samples of *C. proliferus* between 0.5 and 5 cm in diameter were obtained by trimming the plants. In the cases of wheat and barley straw, these two agricultural residues were provided by Ecopapel S.L. Company (Écija, Spain). Both raw materials were conditioned previously to use in the pulping processes to discard undesirable components such as stones or seeds.

### 3.2. Pulping Conditions

To perform all the pulping processes, a 15-L batch cylindrical reactor (Metrotec^®^, Basque, Spain) that was heated by means of an electrical wire and connected to a rotatory axle to provide a proper agitation was used. This reactor monitored pressure, temperature and cooking time. Operating conditions for *L. leucocephala* and *C. proliferus* were 13% NaOH, 0.1% Anthraquinone (both over dried material), 185 °C, an 8 liquid/solid ratio, and a cooking time of 60 min. In contrast, cereal straws were cooked under the following operating conditions: 7% NaOH (o.d.m.), 100 °C, a 10 liquid/solid ratio, and a cooking time of 150 min. Both processes are illustrated in Figure 1.

### 3.3. Black Liquor Composition

The characterization of the obtained black liquors was determined using different laboratory analyses. The pH was analysed using a Crison GLP 21 pH meter (Crison Instruments, Barcelona, Spain). The density was determined by dividing their total mass by its total volume. The total dry solids (TDS) of these liquors was determined by drying samples in porcelain crucibles at 105 °C for 24 h. Afterwards, the samples were heated in a muffle at 575 °C for 3 h to measure the ash content [39]. A simple mass balance was performed to determine the lignin content of these spent liquors. Additionally, electrical conductivity was analysed with a Crison GLP 31 conductivity meter (Crison Instruments). All analytical measurements were performed in triplicate.

An aliquot of the different liquors was subjected to quantitative posthydrolysis with 4% H_2_SO_4_ at 121 °C for 60 min to determine its monosaccharides, acetic acid, furfural and hydroxymethylfurfural (HMF) content. Afterwards, the resulting supernatant was filtered through a 0.45-µm membrane filter before high-performance liquid chromatography (HPLC) analysis. HPLC analyses were performed using an Aminex HPX-87H column (Bio-Rad, Berkeley, CA, USA) at 30 °C eluted with 0.01 M H_2_SO_4_ at a flow rate of 0.6 mL∙min^−1^ using a refractive index (RI) detector to quantify glucose, xylose, arabinose, acetic acid, HMF and furfural [49].

### 3.4. Lignin Isolation

Lignin isolation was performed by precipitating the dissolved lignin from all the spent liquors by acidifying them to a pH of 2. Acid precipitation of the obtained liquors was made using a concentrated solution (95%) of sulfuric acid. After lowering the pH, solutions were kept for 24 h to allow the sedimentation of the precipitated lignin. The next steps were to centrifuge the samples (8000 rpm for 20 min) and wash them with distilled water twice to discard possible impurities such as sugars or inorganic particles; finally, the samples were dried at 60 °C in an oven for 48 h. The scheme of this process is presented in Figure 1.

### 3.5. Lignin Fraction Characterization

Lignin fractions were analysed with the purpose of establishing their physicochemical characteristics. For lignin composition, the ash content was determined in duplicate with the same procedure explained in the black liquor composition section but with some modifications. In this case, samples were heated to 800 ± 25 °C and maintained at this temperature until the absence of black particles [50]. Also, the dry weight of the samples was previously measured by drying them in weighed porcelain crucibles at 105 °C for 24 h.

To determine the Klason lignin and acid soluble lignin, an acid hydrolysis was performed in two steps: (1) 300 mg of each sample was subjected to an acid hydrolysis with (72% *w/w*) H_2_SO_4_ at 30 °C for 1 h; and (2) the resulting mixture was diluted with deionized water to obtain a 4% H_2_SO_4_ solution and heated in an autoclave to 121 °C for 60 min. Final solutions were filtered through a sintered glass crucible (number 3), previously weighed. Crucibles were then washed twice and dried in an oven at 105 °C for 24 h to determine the Klason lignin content. The hydrolysate solutions were analysed to measure acid soluble lignin (ASL, UV-absorption at 205 nm) and monomeric sugars. This last analysis was performed using a method that has been reported before [49]. No correction factors for possible sugar degradation during the two-step acid hydrolysis of lignin precipitates were used.

A thermogravimetric analysis (TGA) was performed to study the thermal properties. Measurements were performed using a TGA/DSC-1 thermobalance (Mettler Toledo, Greifensee, Switzerland) in an inert nitrogen atmosphere. Scans were run from 30 to 800 °C with a heating rate of 10 °C/min, and each sample weighed approximately 10 mg.

Attenuated total reflectance infrared Fourier transform spectroscopy (ATR-IR). The infrared (IR) spectra of the lignin samples were recorded using a Spectrum Two^TM^ instrument (Perkin Elmer, Waltham, MA, USA) by the attenuated total reflectance (ATR) technique. The spectra were recorded from 4000 to 450 cm^−1^ with a resolution of 4 cm^−1^ and performing 20 scans.

Heteronuclear single-quantum correlation (HSQC) nuclear magnetic resonance spectroscopy. A 400 MHz Bruker Avance spectrometer (‎Billerica, MA, USA), equipped with a 5-mm Bruker Inverse Broad Band (BBI) probe, working at ^1^H frequency of 400.13 MHz, was employed to conduct the liquid-state NMR measurements at a temperature of 298 ± 1 K. Lignin samples (around 7 mg) were dissolved in deuterated DMSO (700 μL) and placed in 5-mm NMR tubes. HSQC (Heteronuclear Single-Quantum Correlation) experiments were acquired to examine the molecular composition of lignin sub-units. Each HSQC was composed of 512 experiments (F1) and 2048 acquisition points (F2). The spectral widths were 16 (6410.3 Hz) and 250 (25,155.7 Hz) ppm for ^1^H and ^13^C nuclei, respectively. The 2D spectra consisted of 16 dummy scans, 80 total transients and 0.5 μs of trim pulse length. The optimal value of 145 Hz, as ^1^H-^13^C scalar J-coupling, was accounted for to optimize the acquisition parameters. The centre of the solvent peak was used as an internal reference (*δ*C/*δ*H 39.5/2.49) to calibrate the frequencies axes.

### 3.6. Solid-State ^13^C-Cross Polarization Magic Angle Spinning Nuclear Magnetic Resonance (NMR) Spectroscopy

The solid-state ^13^C NMR spectra were acquired with a 300 MHz (7.0 Tesla) Bruker Avance magnet (Bruker Bio Spin GmbH, Rheinstetten, Germany), composed of a wide-bore system and equipped with a CPMAS (Cross-Polarization Magic-Angle-Spinning) probe, working at ^13^C frequency of 75.47 MHz. Samples were loaded into 4-mm zirconia rotors, closed with Kel F caps and spun at a rate of 10,000 ± 1 Hz. Such spectra were acquired by applying a cross-polarization technique and consisted of 1814 time domain points, a spectral width of 300 ppm (22,727.3 Hz), a recycle delay of 2 s, 5000 scans and 1 ms of contact time. The ^13^C-CPMAS pulse sequence was conducted by using a 1H Ramp pulse to account for the non-homogeneity of the Hartmann–Hahn condition. A TPPM15 scheme was applied to perform the ^13^C-^1^H decoupling. The Free Induction Decay (FID) was transformed by applying a 4k zero filling and an exponential filter function with a line broadening of 100 Hz. All spectra were processed using MestReC NMR Processing Software (v. 4.9.9.9, (Mestrelab Research, Santiago de Compostela, Spain).

## 4. Conclusions

In this paper, the physico-chemical properties of four lignin samples extracted from different raw materials (wheat straw, barley straw, *L. leucocephala* and *C. proliferus*) by an alkali extraction method have been characterized. The 2D-NMR revealed that all the lignins studied were HGS type, which is mainly due to the raw biomasses used. However, the same analysis showed differences between the wheat straw lignin and the other three samples in terms of aromatic units and inter-unit linkages. These three lignins presented an identical chemical structure and similar thermal behaviour and chemical composition. This is an important fact given that lignins usually have different composition and structure depending on the botanical origin and the extraction method used. These three lignin samples were obtained from diverse agricultural residues, which could be very attractive from an industrial point of view—obtaining the same product using dissimilar starting materials. Conversely, wheat straw lignin had a higher Klason lignin content but a lower carbohydrates content compared with the other three samples. The latter is reflected in the thermal behaviour, with the wheat straw lignin having a greater thermal stability. Moreover, there are some molecular structures whose signals were only detected in this wheat straw lignin. Such compounds as *p*-coumarate or ferulate could be extracted from this lignin and used in certain industries as a chemical product. The comprehensive study of the composition and chemical structure of lignins obtained from spent liquors in the paper industry will improve biorefinery operations and help provide different products for green chemistry applications. This could improve the environmental and economic value of biorefineries.

## Figures and Tables

**Figure 7:**
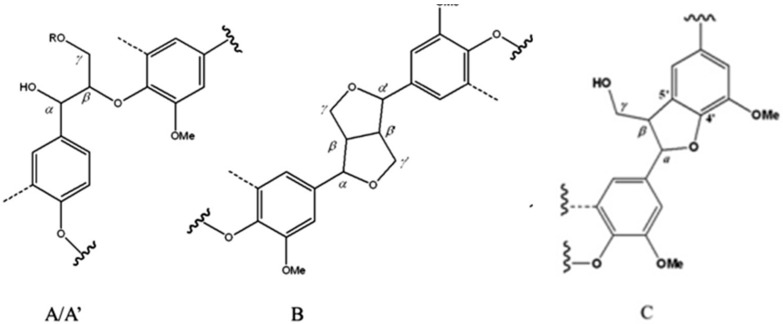

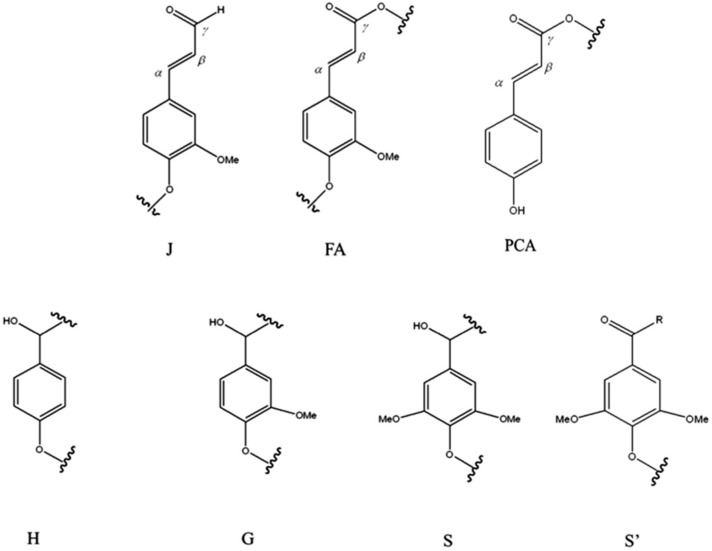
Main molecular structures identified in wheat and *Chamaecytisus proliferus* lignin extracts by ^13^C-^1^H 2D-HSQC NMR spectroscopy. (**A**) β-*O*-4′ linkages; (**A′**) β-*O*-4′ linkages with acetylated γ-carbon; (**B**) resinol β*-*β′ structure; (**C**) phenylcoumaran β-5′ subunit; (**H**) *p*-hydroxyphenyl unit; (**G**) guaiacyl unit; (**S**) syringyl unit; (**S′**) oxidized syringyl units with a C_α_ ketone; (**FA**) ferulate; (**PCA**) *p*-coumarate and (**J**) cinnamyl aldehyde end-groups.

**Table 1:** Physicochemical properties of the black liquors derived from the different raw materials.

Parameters	Soda Wheat Straw BL ^1^	Soda Barley Straw BL ^1^	Soda-AQ ^3^ *Leucaena* BL ^1^	Soda-AQ ^3^ *Chamaecytisus* BL ^1^
pH	10.72 ± 0.16	10.24 ± 0.011	9.85 ± 0.005	10.47 ± 0.006
Density (g/mL)	1.011 ± 0.002	1.004 ± 0.007	1.021 ± 0.002	1.017 ± 0.003
Total Dry Solids (g/L)	32.57 ± 0.13	45.67 ± 0.21	55.80 ± 0.21	51.74 ± 0.93
Ash (g/L)	15.99 ± 0.42	16.77 ± 1.22	23.69 ± 0.33	20.84 ±1.60
Lignin (g/L)	11.6	12.6	24.3	19.0
Total monosaccharides (g/L)	3.812	4.004	4.239	5.049
Glucose (g/L)	0.127	0.211	1.237	0.262
Xylose (g/L)	1.682	2.814	1.781	2.624
Arabinose (g/L)	0.706	0.240	0.428	0.649
Acetyl groups (g/L)	1.297	0.739	0.792	1.514
HMF ^2^ (g/L)	0.00	0.00	0.00	0.00
Furfural (g/L)	0.00	0.00	0.00	0.00
Electrical conductivity (mS/cm)	14.63 ± 0.01	13.007 ± 0.005	21.7 ± 0.42	21.4 ± 0.06

^1^ Black liquors; ^2^ Hydroxymethylfurfural; ^3^ Anthraquinone.

**Table 2:** Yield and composition of lignin samples.

Sample	Yield of Precipitated Fractions		Yield as Pure Lignin (% Lignin/BL ^1^)	Chemical Composition of Precipitated Fractions			
	(g/L BL ^1^)	As Pure Lignin (g/L BL ^1^)		KL ^2^	ASL ^3^	Carb ^4^	Ash
				(% of o.d.m.)			
Wheat straw	18.25	12.56	108.28	64.90	3.91	2.76	29.25
Barley straw	18.48	9.93	78.81	49.46	4.25	12.27	18.45
*Leucaena*	26.62	14.66	60.33	47.74	7.34	11.44	19.47
*Chamaecytisus*	22.95	13.95	73.42	52.23	8.56	12.25	15.59

^1^ Black liquors; ^2^ Klason lignin; ^3^ Acid soluble lignin; ^4^ Carbohydrates.

**Table 3:** Thermogravimetric parameters of the different lignin samples.

Sample	T_onset_ (°C)	DTG_max_ (°C)	*T*_50_% (°C)	Residue (%)
^1^ WS_Soda Lignin	220	342/−0.1339	780	46.1
^2^ BS_Soda Lignin	207	310/−0.1796	552	42.7
^3^ CP_Soda-AQ Lignin	209	310/−0.1811	548	46.0
^4^ LL_Soda-AQ Lignin	208	320/−0.1732	672	47.0

^1^ Wheat straw; ^2^ barley straw; ^3^ *Chamaecytisus proliferus*; ^4^ *Leucaena leucocephala*.

**Table 4:** Carbon distribution (%) in different chemical shift regions (ppm) in ^13^C-CPMAS-NMR spectra of lignins from ^1^ *Chamaecytisus* and wheat.

Range (ppm)	*Chamaecytisus*	Wheat
0–40 (alkyl groups)	15.2	22.7
40–110 (*O*-alkyl, carbohydrates)	54.1	49.2
110–160 (aromatics and phenolics)	27.0	23.8
160–200 (carbonyl and carboxyl groups)	3.4	4.3
^2^ HB	42.2	46.5
^3^ HI	57.5	53.6
HB/HI	0.73	0.87
Alkyl-related ^4^ hydrophobicity index	0.36	0.49

^1^ Since the ^13^C-CPMAS spectra of lignins isolated from *Chamaecytisus*, *Leucaena* and barley were not significantly different (Figure S1), the integration refers to the spectrum from *Chamaecytisus*; ^2^ Sum of integration of signals between 0–40 and 110–160 ppm; ^3^ Sum of integration of signal between 40–110 and 160–200 ppm; ^4^ Integration of signals between 0–40 divided by the sum of the area of signals between 0–40 and 110–160 ppm.

**Table 5:** Assignment of main ^13^C-^1^H correlation signals in HSQC spectra of lignin from wheat and *Chamaecytisus* ^a^ shown in Figure 6.

Label	δC	δH	Assignment
B_β_	53.4	3.1	C_β_-H_β_ in resinol substructures (B)
OMe	55.5	3.7	C-H in methoxy groups
Aγ	59.3	3.2 and 3.9	C_γ_-H_γ_ in β-*O*-4′ substructures (A)
A′γ	62.9	3.9	C_γ_-H_γ_ in γ-acylated β-*O*-4′ substructures (A′)
X_5_	63.0	3.2	C_5_-H_5_ in β-xylopyranoside
Aα (G)	69.4	4.5	C_α_-H_α_ in β-*O*-4′ substructures (A) linked to a G-unit
Bγ	70.6	3.3 and 3.9	Cγ-Hγ in resinol substructures (B)
Cγ	70.9	4.2	Cγ-Hγ in β-5′ phenylcoumaran subunit (C) ^c^
A′α (G)	71.1	4.8	C_α_-H_α_ in γ-acylated β-*O*-4′ substructures linked to a G-unit (A′) ^c^
X′_2_	72.4	4.9	C_2_-H_2_ in 2-*O*-Ac-β-d-xylopyranoside ^c^
X_2_	72.5	3.0	C_2_-H_2_ in β-xylopyranoside
X_3_	73.8	3.3	C_3_-H_3_ in β-xylopyranoside
X_4_	75.3	3.5	C_4_-H_4_ in β-xylopyranoside
U_4_	81.6	3.1	C_4_-H_4_ in 4-*O*-methyl-α-d-glucuronic acid
B*α*	85.1	4.6	C_α_-H_α_ in resinol (β-β) substructures (B)
Aβ (S)	85.9	4.1	C_β_−H_β_ in β-*O*-4′ substructures (A) linked to a S-unit ^c^
S_2,6_	103.9	6.7	C_2,6_-H_2,6_ in etherified syringyl units (S)
S′_2,6_	106.8	7.2	C_2,6_-H_2,6_ in oxidized (C_α_=O) phenolic syringyl units (S′)
FA_2_	110.6	7.4	C_2_-H_2_ in ferulate (FA) ^c^
G_2_	111.7	6.6	C_2_-H_2_ in guaiacyl units (G)
J_2(G)_	112.1	7.4	C_2_-H_2_ in cinnamyl aldehyde end-groups (J) ^c^
H_3,5_	115.1	6.7	C_3,5_-H_3,5_ in *p*-hydroxyphenyl units (H)
G_5_	115.6	6.7	C_5_-H_5_ in guaiacyl units (G)
PCA_3,5_	117.1	6.4	C_3,5_-H_3,5_ in *p*-coumarate PCA and ferulate (FA) ^c^
G_6_	119.8	6.8	C_6_-H_6_ in guaiacyl units (G) ^c^
J_6(G)_	122.7	7.1	C_6_-H_6_ in cinnamyl aldehyde end-groups (J) ^c^
FA_6_	122.9	7.1	C_6_-H_6_ in ferulate (FA) ^c^
H_2,6_	129.0	7.2	C_2,6_-H_2,6_ in *p*-hydroxyphenyl units (H) ^c^
PCA_2,6_	130.3	7.5	C_2,6_-H_2,6_ in *p*-coumarate (PCA) ^c^
PCAα and FAα	144.5	7.5	C_α_-H_α_ in *p*-coumarate (PCA) and ferulate (FA) ^c^
Anomeric Region			
αX1 (R)	4.9	92.2	α-d-Xylopyranoside (R) [α-d-glucopyranoside (R)]
U_1_	5.1	97.2	4-*O*-Methyl-α-d-glucuronic acid ^b^
βX1 (R)	4.2	97.3	β-d-Xylopyranoside (R) [β-d-glucopyranoside (R)]
UGA_1_	4.9	101.7	Methyl(1/4)-α-d-galacturonate
M_1_	4.5	101.0	(1/4)-*β*-d-Mannopyranoside ^b^
X_1_-X′_1_	4.3	101.5	β-d-Xylopyranoside + 3-*O*-acetyl-β-d-xylopyranoside

^a^ Since the ^13^C-^1^H HSQC spectra of lignins isolated from *Chamaecytisus*, *Leucaena* and barley lignins were not significantly different (Figure S2), the interpretation refers to HSQC spectrum from *Chamaecytisus*; ^b^ Not present in Wheat-derived lignin; ^c^ Not present in *Chamaecytisus*-derived lignin.

## Supplementary Materials

Supplementary materials can be found at www.mdpi.com/1422-0067/18/2/327/s1.
