# Supplementary material for: Isolation and Characterization of Gramineae and Fabaceae Soda Lignins
Source: Int J Mol Sci. 2017 Feb 4;18(2):327. doi: 10.3390/ijms18020327 (PMC5343863; doi:10.3390/ijms18020327)
Supplement: Supplementary file 1 [file ijms-18-00327-s001.pdf]

Int. J. Mol. Sci. **2017**, *18*, 327; doi:10.3390/ijms18020327

Supplementary Materials: Isolation and Characterization of *Gramineae* and *Fabaceae* Soda Lignins

Juan Domínguez-Robles, Rafael Sánchez, Eduardo Espinosa, Davide Savy, Pierluigi Mazzei, Alessandro Piccolo and Alejandro Rodríguez

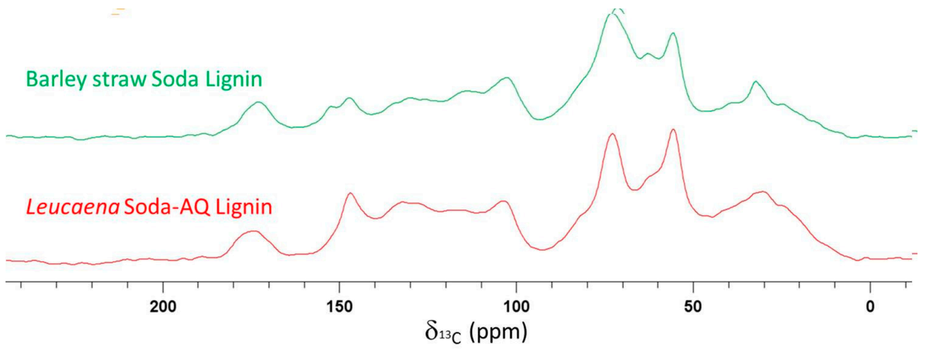

Figure S1. ^{13}C-CPMAS solid-state NMR spectra of lignin samples from barley straw and *Leucaena leucocephala* lignin extracts.

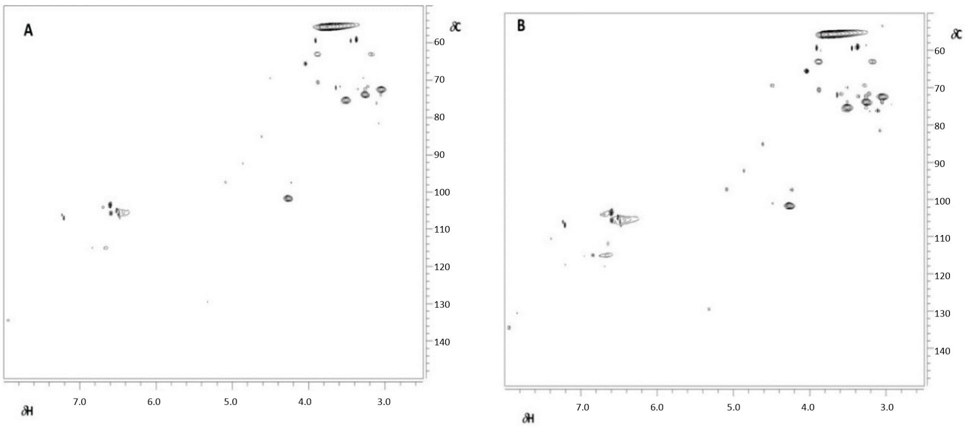

Figure S2. ^{13}C-^{1}H 2D-HSQC NMR spectra of lignins isolated from barley straw (**A**) and *Leucaena leucocephala* (**B**).

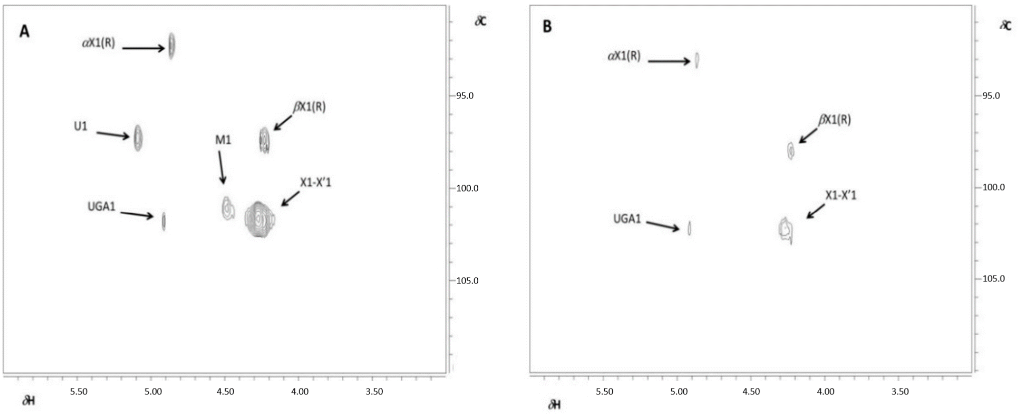

Figure S3. Anomeric part of the HSQC spectra of lignins isolated from *Chamaecytisus proliferus* (**A**) and wheat straw (**B**), respectively. The anomeric parts of the HSQC spectra of lignins isolated from *Leucaena leucocephala* and barley straw are the same as those presented for *Chamaecytisus proliferus*.
